# Is noise-induced hearing loss still a public health problem after decades of legislation?^☆^

**DOI:** 10.1016/j.bjorl.2020.04.001

**Published:** 2020-04-10

**Authors:** Vagner Antonio Rodrigues da Silva, Edson Ibrahim Mitre, Agrício Nubiato Crespo

**Affiliations:** a Universidade Estadual de Campinas (UNICAMP), Faculdade de Ciências Médicas, Departamento de Oftalmo e Otorrinolaringologia, Campinas, SP, Brazil; b Faculdade de Ciências Médicas da Santa Casa de São Paulo, Departamento de Otorrinolaringologia, São Paulo, SP, Brazil

Exposure to noise is a common and the most preventable cause of hearing loss. Occupational noise is responsible for approximately 16% of disabling hearing loss in adults. Noise-Induced Hearing Loss (NIHL) is the second most common occupational illness or injury, even after decades of study, regulation and interventions in the workplace to try to prevent it.^1^

NIHL is irreversible, manifesting first and predominantly at the frequencies of 3 kHz, 4 kHz and 6 kHz. It takes longer for the frequencies of 0.25 kHz, 0.5 kHz, 1 kHz, 2 kHz and 8 kHz to be compromised.^1^ It rarely leads to profound hearing loss. It generally does not exceed 40 dB HL at low frequencies and 75 dB HL at high frequencies. After exposure to noise has ceased, NIHL does not progress.^1^, ^2^

NIHL etiopathogenesis is a multifactorial one. Several genes are involved in different mechanisms that increase susceptibility to noise.^3^ The noise can induce an excessive production of free radicals that remain active for up to seven days inside the cochlea. There is a reduction in intracellular Adenosine Triphosphate (ATP) caused by sustained activation of AMP-Activated Protein Kinase (AMPK), leading to cell apoptosis. Proteins such as caspase 3 and c-Jun N-terminal kinase (JNK) can be activated by noise and cause apoptosis. An excessive accumulation of intracellular calcium can also initiate the oxidative stress chain. Another known phenomenon is synaptopathy, which is the loss of connections between the inner hair cells and their afferent neurons, resulting from glutamate excitotoxicity at postsynaptic terminals.^1^

Risk awareness must be associated with patient motivation to take the necessary measures to reduce exposure to potentially harmful noise, both in the workplace and during leisure activities.^4^ Headphones can produce a sound pressure of up to 126 dB HL. Ambient noise at nightclubs and concerts is another common source of leisure noise that range from 104.3–112.4 dB HL, with an average level of 97.9 dB HL.^5^

Listening to music during sports activities increases attention, reduces fatigue and increases alertness. The effects obtained on performance depend on the characteristics of the stimulus, the individual and the intensity and type of activity. The more pleasant a song is, the louder you want to hear it.

The appropriate frequency of occupational audiometric tests is poorly studied, and it is not based on population studies. There is general medical agreement that it audiometry should be performed annually. Brazilian legislation is the only one that establishes that the first periodic audiometric examination must be performed after six months of work, becoming annual as of the second periodic examination.

Although the improvement in the standards and control of exposure to noise have advanced since the end of World War II, NIHL remains a significant public health problem. The problems caused by NIHL go beyond the auditory symptoms and may affect the vestibule, which increases the risks of accidents in the workplace, in addition to insomnia, irritability and arterial hypertension, decreasing productivity and resulting in risks to the workers’ health. There is much to be done to prevent the condition in populations that are genetically at greater risk for NIHL. The popularization of headphones to listen to music has resulted in early sensorineural hearing loss in young individuals and adolescents. It makes it difficult to identify workers whose audiometric thresholds have worsened exclusively due to their professional activity.

## Conflicts of interest

The authors declare no conflicts of interest.

## References

[1] Lie A., Skogstad M., Johannessen H.A., Tynes T., Mehlum I.S., Nordby K.C. (2016). Occupational noise exposure and hearing: a systematic review. Int Arch Occup Environ Health.

[2] Nordmann A.S., Bohne B.A., Harding G.W. (2000). Histopathological differences between temporary and permanent threshold shift. Hear Res.

[3] Konings A., Van Laer L., Van Camp G. (2009). Genetic studies on noise-induced hearing loss: a review. Ear Hear.

[4] John G.W., Grynevych A., Welch D., McBride D., Thorne P.R. (2014). Noise exposure of workers and the use of hearing protection equipment in New Zealand. Arch Environ Occup Health.

[5] Van Dyck E. (2019). Corrigendum: Musical intensity applied in the sports and exercise domain: an effective strategy to boost performance?. Front Psychol.

